# Antioxidant Responses Induced by Short-Term Activity–Estivation–Arousal Cycle in *Pomacea canaliculata*

**DOI:** 10.3389/fphys.2022.805168

**Published:** 2022-02-02

**Authors:** Maximiliano Giraud-Billoud, Alejandra D. Campoy-Diaz, Federico A. Dellagnola, Cristian Rodriguez, Israel A. Vega

**Affiliations:** ^1^IHEM, CONICET, Universidad Nacional de Cuyo, Mendoza, Argentina; ^2^Facultad de Ciencias Médicas, Instituto de Fisiología, Universidad Nacional de Cuyo, Mendoza, Argentina; ^3^Departamento de Ciencias Básicas, Escuela de Ciencias de la Salud-Medicina, Universidad Nacional de Villa Mercedes, San Luis, Argentina; ^4^Departamento de Biología, Facultad de Ciencias Exactas y Naturales, Universidad Nacional de Cuyo, Mendoza, Argentina

**Keywords:** hypometabolism, oxidative stress, preparation for oxidative stress, redox-sensitive transcription factors, apple snails (*Pomacea* spp.)

## Abstract

Long-term estivation (45 days) in the apple snail *Pomacea canaliculata* induces an increase of non-enzymatic antioxidants, such as uric acid and reduced glutathione (GSH), which constitutes an alternative to the adaptive physiological strategy of preparation for oxidative stress (POS). Here, we studied markers of oxidative stress damage, uric acid levels, and non-enzymatic antioxidant capacity, enzymatic antioxidant defenses, such as superoxide dismutase (SOD), catalase (CAT), and glutathione S-transferase (GST), and transcription factors expression [forkhead box protein O (FOXO), hypoxia-inducible factor-1 alpha (HIF1α), and nuclear factor erythroid 2-related factor 2 (Nrf2)] in control active animals, 7-day estivating and aroused snails, in digestive gland, gill, and lung tissue samples. In the digestive gland, SOD and CAT activities significantly increased after estivation and decreased during arousal. Meanwhile, GST activity decreased significantly during the activity–estivation–arousal cycle. Gill CAT activity increased significantly at 7 days of estivation, and it decreased during arousal. In the lung, the CAT activity level increased significantly during the cycle. FOXO upregulation was observed in the studied tissues, decreasing its expression only in the gill of aroused animals during the cycle. HIF1α and Nrf2 transcription factors decreased their expression during estivation in the gill, while in the lung and the digestive gland, both transcription factors did not show significant changes. Our results showed that the short-term estivation induced oxidative stress in different tissues of *P. canaliculata* thereby increasing overall antioxidant enzymes activity and highlighting the role of FOXO regulation as a possible underlying mechanism of the POS strategy.

## Introduction

Several organisms live under harsh environmental conditions and therefore have evolved different strategies to cope with them. Particularly during hypometabolic situations where the imbalance of oxidative stress can damage self-cells or tissues, many animals show physiological protective strategies known collectively as “preparation for oxidative stress (POS)” (Hermes-Lima et al., 1998, 2015; Giraud-Billoud et al., 2019). Although, the evidence has confirmed the POS strategy in more than 80 animal species from eight different phyla: Cnidaria, Nematoda, Annelida, Tardigrada, Arthropoda, Mollusca, Echinodermata, and Chordata (Moreira et al., 2016), the underlying mechanisms are still not fully understood.

Molecular mechanisms putatively involved in the POS strategy include (a) DNA methylation and histone modifications, (b) regulation of transcription factors, (c) control of mRNA translation by microRNAs, and (d) post-translational modifications of antioxidant enzymes (Giraud-Billoud et al., 2019). In particular, antioxidant response elements (ARE) are cytoprotective genes, which are upregulated under situations of high levels of reactive oxygen species (ROS) and electrophilic compounds, in order to reduce the damage to intracellular macromolecules that could lead to cell death (Pamplona and Costantini, 2011). Under hypoxia, the upregulation of genes like forkhead box protein O (FOXO), nuclear factor erythroid 2-related factor 2 (Nrf2), and hypoxia-inducible factor-1 alpha (HIF1α), among others, has been described (Kobayashi and Yamamoto, 2006; Webb et al., 2009; Malik and Storey, 2011). These REDOX-sensitive transcription factors cause an increase of endogenous antioxidants to cope with ROS overproduction (Ensminger et al., 2021). FOXO regulates expression of target genes controlling different cell responses like stress tolerance, and inducing, for example, an increase of mRNA and concentration of superoxide dismutase (SOD), and it regulates catalase (CAT) expression during hypometabolic situations (Kops et al., 2002; Malik and Storey, 2011; Ponugoti et al., 2013). HIF1α is expressed in response to hypoxia and activates antioxidants such as SOD, CAT, glutathione S-transferase (GST), and glutathione peroxidase (Song et al., 2015; Lacher et al., 2018). Nrf2 is a transcription factor that is negatively regulated by Kelch-like ECH-associated protein 1 (Keap1). Electrophile molecules and ROS may modify cysteine residues in Keap1, which triggers transcriptional regulation of antioxidant proteins throughout Nrf2 stimulation. These include SOD, CAT, GST, and other proteins involved in scavenging ROS and reduced glutathione (GSH) biosynthesis (Zhu et al., 2005; Hayes and McMahon, 2009; Kovac et al., 2015; Suzuki and Yamamoto, 2015). Changes in the expression of these genes have not been studied until now in the context of estivation in mollusks. In order to evaluate this role and to shed light on the molecular physiology of this phenomenon, we have established a short-term experimental model of activity–estivation–arousal cycle in the apple snail *Pomacea canaliculata* (Caenogastropoda, Ampullariidae).

Apple snails are a conspicuous clade of amphibious snails that show broad adaptive capacities to survive extreme environmental conditions such as desiccation, cold, or high salinity (Giraud-Billoud et al., 2013, 2018; Hayes et al., 2015; Yang et al., 2018). In particular, *P. canaliculata* develops a peculiar form of POS when it is exposed to prolonged periods (45 days) of environmental stress (estivation or hibernation; Giraud-Billoud et al., 2013, 2018). After long-term estivation, non-enzymatic antioxidant defense mechanisms (e.g., increased circulating levels of GSH and uric acid) allow arousing safely from the hypometabolic state, restoring the physiological conditions of active animals within 24 h of re-immersion in water (Giraud-Billoud et al., 2011, 2013). Besides, proteomic studies in this species have shown that CAT expression increases after a 30-day-period of estivation (Sun et al., 2013). These findings suggest that, in the initial stages of the activity–estivation–arousal cycle, *P. canaliculata* prepares for oxidative stress through activating enzymatic antioxidant defense mechanisms, as is shown for other animal species (Hermes-Lima et al., 2015). Thus, a plausible hypothesis to test is that, once antioxidant enzymatic defenses are diminished by molecular or cellular alterations induced by stress, non-enzymatic defense mechanisms would play a leading role in tissue protection.

Attempting to answer this question, we evaluated the physiological response at the tissue level, including the expression of REDOX-sensitive transcription factors, in a model of a short-term (7 days) activity–estivation–arousal cycle in *P. canaliculata*. Thus, we aimed (a) to determine the production of ROS and the consequent damage to macromolecules from oxidative stress, and (b) to evaluate the participation of non-enzymatic and enzymatic antioxidant defense mechanisms during the cycle. In addition, we (c) assessed for changes in the expression of REDOX-sensitive transcription factors (FOXO, HIF1α, and Nrf2) as potential molecular mechanisms that lead to the development of the POS strategy during the activity–estivation–arousal cycle.

## Materials and Methods

### Animals

The adult animals (both sexes) used in all experiments came from our laboratory strain (stock origin and culturing conditions have been previously reported, Giraud-Billoud et al., 2013). Procedures for snail culture, sacrifice, and tissue sampling were approved by the Institutional Committee for the Care and Use of Laboratory Animals (CICUAL, Facultad de Ciencias Médicas, Universidad Nacional de Cuyo), Approval Protocol No 55/2015.

### Short-Term Activity–Estivation–Arousal Cycle Induction and Tissue Sampling

Experimental groups comprised by six adult animals were allotted to the following categories: (1) active control snails (Ctrl); (2) estivated snails for 7 days (Est); and (3) aroused snails (Ar), 20 min after the operculum was detached from the shell aperture following water exposure (Giraud-Billoud et al., 2011).

Tissue samples from the gill, lung, and digestive gland (midgut gland or hepatopancreas) were dissected, immediately frozen in liquid nitrogen, and stored at −80°C until use.

### ROS Production

Reactive oxygen species production was evaluated according to Wang and Joseph (1999). Tissue samples were homogenized (1:5 w/v) in 100 mM Tris-HCl buffer with 5 mM MgCl_2_ and 2 mM EDTA. Homogenates were centrifuged at 10,000*g* (4°C) for 20 min, and the supernatants were incubated with 40 mM 2′,7′-dichlorofluorescein diacetate (DCFH-DA) in buffered solution (30 mM HEPES, 200 mM KCl, and 1 mM MgCl_2_) for 20 min (37°C). The released DCFH-DA was oxidized by ROS, forming a fluorescent compound, DCF, which was excited at 485 nm and detected at 538 nm. Results were expressed as arbitrary units of florescence (AUF) per milligram of wet tissue per minute (AUF/mg/min).

### Hypoxic Damage Markers

Protein oxidative damage (concentration of carbonyl groups, CG) was determined as described by Levine et al. (1990), based on the addition of carbonyl group to 2,4-dinitrophenylhydrazine (DNPH). Tissues were homogenized in 20 mM potassium phosphate (pH 7.4) and then centrifuged at 10,000*g* for 30 min. The supernatants containing proteins were incubated for 60 min in a solution of 2 M HCl, 10 mM DNPH, this solution was then precipitated with 20% (w/v) trichloroacetic acid. After that, the precipitates were washed with ethanol/ethyl acetate (1:1) and dissolved in a solution of 20 mM potassium phosphate (pH 2.3) containing 6 M guanidine hydrochloride. CG content was determined by measuring the absorbance at 360 nm (Reznick and Packer, 1994). Results were expressed as nanomole of carbonyl groups per milligram of protein (nmol/mg). Protein concentration was measured according to the method of Bradford (1976).

Thiobarbituric acid reactive substances (TBARS) were quantified as an index of lipid peroxidation. Tissue samples (~100 mg) were homogenized (Ultraturrax® homogenizer) in 900 μl of 0.1 M sodium phosphate buffer (pH 7.0) and centrifuged (10,500*g*, 5 min), and the supernatants were kept frozen until TBARS quantification with the method described by Wasowicz et al. (1993) and modified by Lapenna et al. (2001). The aliquots were mixed with 1 ml of working solution (15% w/v trichloroacetic acid, 0.25 M hydrochloric acid, 0.67% w/v thiobarbituric acid, 2.25 mM butylated hydroxytoluene solution, and 0.1 ml of 8.1% SDS) and kept at 4°C during the process. After that, the samples were heated at 95°C for 30 min and 3 ml of butanol were added. Finally, tubes were stirred for 5 min and centrifuged at 1,500*g* for 10 min. Organic layers were collected and placed in glass cuvettes and TBARS were spectrophotometrically determined at 540 nm, with an extinction coefficient of 156 mM^−1^. The concentration was expressed as nanomole of TBARS per gram of wet tissue (nmol/g). Lactate concentration was used as an anaerobic glycolysis marker and was measured using a commercial kit (Wiener Lab), following the manufacturer’s instructions. Concentrations of lactic acid were expressed as micromole per milligram of wet tissue (umol/mg).

### Antioxidant Profile Characterization

Free radical scavenging capacity (oxidation of 2,2′-azino-bis-3-ethylbenzothiazoline-6-sulfonic acid radical, ABTS^+^), concentration of soluble uric acid as non-enzymatic antioxidant, antioxidant enzymes activity (SOD, CAT, and GST), and proteins were measured as antioxidant profile characterization. Frozen tissue samples (approximately 100 mg) were processed using an UltraTurrax® homogenizer in a buffered solution (20 mM Tris-HCl, 1 mM EDTA, 0.15 mM KCl, 1 mM dithioerythritol, 0.5 M sucrose, 0.1 mM phenylmethylsulfonyl fluoride, and pH 7.6), supplemented with Halt™ Protease Inhibitor Cocktail (Thermo Fisher) and then centrifuged 30 min at 4°C (10,500*g*). Supernatants were collected, aliquoted, and frozen until use.

Oxidation of ABTS^+^ was measured by the method of Miller and Rice-Evans (1997). Briefly, in presence of persulphate anions, a colorless salt generates the greenish-blue cationic radical ABTS^+^, which decolorizes when reacts with antioxidants in the sample, hence extinguishing spectrophotometric reading at 734 nm. An ascorbic acid standard curve was used (Giraud-Billoud et al., 2018), and results were expressed as percent ABTS^+^ oxidation.

Uric acid concentration was measured in 100 μl aliquots sample, measuring the amount of hydrogen peroxide formed after treatment with urate oxidase. A colored quinoneimine product generated was quantified at 510 nm, according to Trinder (1969). Uric acid concentration was expressed as millimole of compound per milligram of protein (mmol/mg).

Superoxide dismutase activity was determined by the method described by McCord and Fridovich (1969), where the compound formazan red is formed from mixing xanthine and the enzyme xanthine oxidase, as generators of O_2_^−^ and 2-(4-iodophenyl)-3-(4-nitrophenol)-5-phenyltetrazolium (INT) chloride, which reacts with O_2_^−^. The activity was quantified as the percentage of inhibition compared to a calibration curve performed with purified SOD. CAT activity was quantified by the method of Aebi (1984), which from decomposition of 10 mM H_2_O_2_ in 50 mM phosphate buffer (pH 7.0) and 20 μl of the tissue extract, the enzyme activity was estimated at 240 nm. GST activity was measured according to Habig et al. (1974). GST determination was carried out at constant temperature (25°C) in homogenization buffer, 50 mM 1-chloro-2,4-dinitrobenzene, and 100 mM reduced glutathione. The increase in absorbance (wavelength 340 nm) was measured every 30 s for 120 s. Results of enzyme activity were expressed as Units of SOD and CAT or milliUnits of GST per milligram of protein (U/mg or mU/mg).

Protein concentration was estimated according to the method of Lowry et al. (1951), using 50 μl of sample, and the colored complex was measured at 690 nm.

All the results were expressed as mean ± SEM (*N* = 6 per group).

### REDOX-Sensitive Transcription Factors Expression

Total RNA was extracted from tissue homogenates of four animals for each experimental group, using a NucleoSpin RNA Set for NucleoZOL (Macherey-Nagel), following the supplier’s recommendations. RNA was quantified (NanoDrop ND-100 spectrophotometer) and stored at −20°C. Expression levels of FOXO, HIF1α, and NRf2 genes were assessed by quantitative RT-qPCR. Around 500 ng of total RNA was used for reverse transcription (M-MLV Reverse transcriptase, Invitrogen Cat.#28025-021). Quantitative PCR was performed in a final volume of 10 μl containing 50 ng of cDNA, iTaq Universal SYBR Green Supermix reaction mix (BIORAD) and 0.5 μM of each specific primer (Supplementary Table 1) using a CFX-96 thermocycler (BIORAD Cat.#1725122). Each specific pair of primers was designed using the genomic information of *P. canaliculata* (Sun et al., 2019). To ensure that amplicons were from mRNA and not from genomic DNA amplification, controls without reverse transcription were included. Validation was performed based on amplicon size and melting point. The relative expression levels of FOXO, HIF1α, and Nrf2 genes in all samples were normalized to β-actin and relative quantification was performed using the 2^−ΔΔCT^ method (Schmittgen and Livak, 2008).

All the results were expressed as relative expression units (REU), each value showed in Table 1 represents mean ± SEM (*N* = 4 per group).

**Table 1:** REDOX-sensitive transcription factors expression in tissues of *P. canaliculata* exposed to short term activity–estivation–arousal cycle.

	Control	Estivation	Arousal
**FOXO gene**			
Gill	0.32 ± 0.12	2.12 ± 0.35^*^	0.28 ± 0.04^**^
Lung	0.72 ± 0.23	61.3 ± 12.5^*^	64.8 ± 14.3^*^
Digestive gland	0.98 ± 0.41	4.90 ± 3.57	1.38 ± 0.37
**HIF1α gene**			
Gill	1.81 ± 0.56	0.58 ± 0.16^*^	0.20 ± 0.14^*^
Lung	1.09 ± 0.27	4.26 ± 1.581	2.29 ± 0.68
Digestive gland	0.82 ± 0.35	2.40 ± 1.21	1.31 ± 0.15
**Nrf2 gene**			
Gill	3.50 ± 1.15	0.11 ± 0.03^*^	0.49 ± 0.16^*^
Lung	1.62 ± 0.31	1.91 ± 0.44	1.12 ± 0.03
Digestive gland	ND	ND	ND

^*^Gene expression as relative expression unit (REU). Each value represents Mean ± SEM. *N* = 4 per group. ND = Not detected.

* Indicates significant differences vs. control group.

** Indicates significant differences between estivation and arousal groups (One-way ANOVA, Newman–Keuls’s post-test).

### Statistical Analysis

For multigroup comparisons, variable distribution was evaluated by Shapiro–Wilk normality test, and equal variance Bartlett’s test was used to evaluate homoscedasticity for each set of experimental variables. Differences among experimental groups (control, estivation, and arousal) and also between tissues (digestive gland, gill, and lung) of each condition of the activity–estivation–arousal cycle were evaluated by one-way ANOVA followed, when significant, with a Newman–Keuls *post hoc* test for multiple comparisons. An ANOVA table with F (Dfn, Dfd) and *p*-values obtained is available in Supplementary Table 2.

## Results

### ROS Production and Oxidative Damage

**Figure 1:**
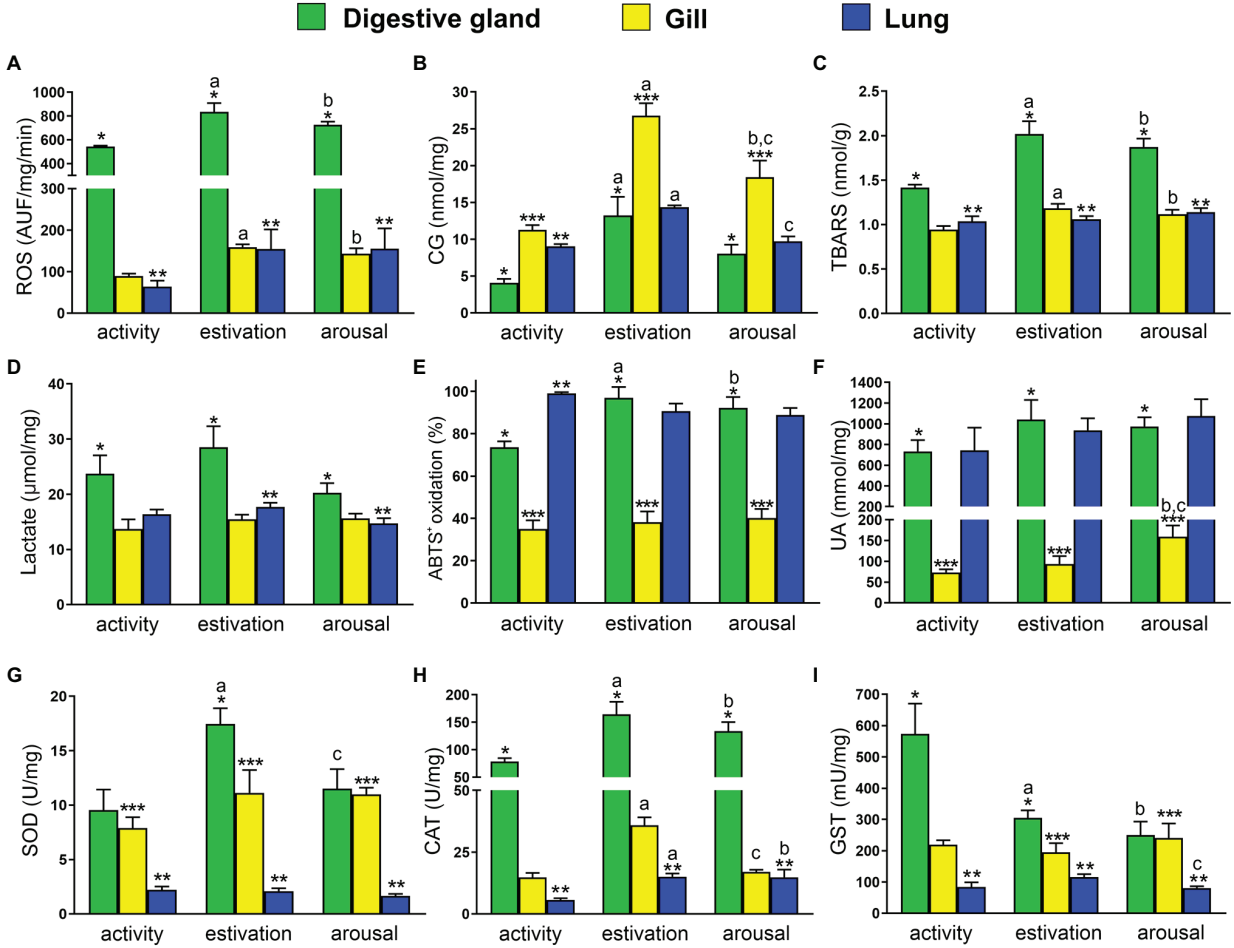
Physiological responses of *Pomacea canaliculata* exposed to short term activity–estivation–arousal cycle. **(A)** Reactive oxygen species (ROS); **(B)** protein oxidative damage (carbonyl groups, CG); **(C)** thiobarbituric acid reactive substances (TBARS); **(D)** lactate; **(E)** percent of ABTS^+^ oxidation; **(F)** non-enzymatic antioxidant (uric acid); **(G–I)** enzymatic antioxidant defenses [superoxide dismutase (SOD), catalase (CAT), and glutathione S-transferase (GST)], in the digestive gland (green), gill (yellow), and lung (blue). Mean ± SEM. Significant differences between activity, estivation, and arousal groups in the same organ (*p* < 0.05, one-way ANOVA, Newman–Keulsʼs test) are indicated as follows: ^a^activity vs. estivation, ^b^activity vs. arousal, ^c^estivation vs. arousal. Significant differences between organs at the same time of the activity–estivation–arousal cycle (*p* < 0.05, one-way ANOVA, Newman–Keulsʼs test) are indicated as follows: * midgut gland vs. gill, ** midgut gland vs. lung, and *** gill vs. lung.

Both estivation-induced ischemia and arousal-induced reperfusion cause an increase in ROS levels. Comparing the studied tissues of each experimental set, the ROS levels in the digestive gland were 7–8-fold higher than the gill and the lung (Figure 1A). On the other hand, the comparison of ROS production between active, estivating, and aroused animals for a same organ, showed that in the digestive gland, ROS levels significantly increased after estivation and arousal, compared to control group (Ctrl = 542.4 ± 8.5; Est = 834.6 ± 74.8; and Ar = 781.0 ± 57.9 AUF/mg/min; Figure 1A). Besides, in the gill, ROS levels almost doubled during 7-day estivation and 20 min of arousal (Ctrl = 89.4 ± 6.1; Est = 159.3 ± 6.5; and Ar = 143.1 ± 13.2 AUF/mg/min; Figure 1A). Also, lung showed an increase of ROS production during estivation (Ctrl = 63.9 ± 14.6; Est = 154.6 ± 47.1; and Ar = 155.4 ± 48.9 AUF/mg/min; Figure 1A).

If the studied tissues had not effective antioxidant protection mechanisms to counteract the ROS overproduction during activity–estivation–arousal cycle, the damage to molecules such as proteins and lipids would have become evident. In the control groups, the levels of CG were 3-fold higher in the gill compared to the digestive gland and 2-fold higher than in the lung (Figure 1B). Protein damage, measured as CG, raised 2-fold in the gill compared to the digestive gland and the lung from estivated and aroused animals (Figure 1B). In the activity–estivation–arousal cycle, the protein damage in the digestive gland increased significantly after estivation (Ctrl = 4.1 ± 0.5; Est = 13.3 ± 2.5; and Ar = 8.1 ± 1.2 nmol/mg; Figure 1B), meanwhile, in the gill a significant increase was observed after estivation and then decreased significantly after the arousal (Ctrl = 11.3 ± 0.6; Est = 26.8 ± 1.7; and Ar = 18.4 ± 2.2 nmol/mg; Figure 1B). Similar significant changes were observed in the lung (Ctrl = 9.0 ± 0.3; Est = 14.4 ± 0.2; and Ar = 9.7 ± 0.7 nmol/mg; Figure 1B). Lipid peroxidation, evidenced by an increase in TBARS levels, was slightly higher in the digestive gland than in the other tissues studied for the control group. However, TBARS levels became more than double in the digestive gland than in the gills and lungs in the estivation and arousal groups (Figure 1C). Furthermore, TBARS concentration of estivating and arousal snails was significantly higher than in control snails, either in the gill (Ctrl = 0.94 ± 0.04; Est = 1.18 ± 0.05; and Ar = 1.12 ± 0.05 nmol/mg; Figure 1C) or the digestive gland (Ctrl = 1.42 ± 0.03; Est = 2.02 ± 0.14; and Ar = 1.87 ± 0.09 nmol/mg; Figure 1C). The lung did not show significant changes in TBARS levels (Figure 1C).

Finally, anaerobic cellular activity induced by hypoxia during estivation was evaluated, because it may induce an increase in lactic acid levels. Lactate concentrations were 2-fold higher in the digestive gland than in the gill and lung of estivating snails, while in the active and aroused groups, although, the values in the digestive gland were significantly higher, the differences between organs were lower. Nonetheless, no significant changes in the lactate concentrations were observed during the activity–estivation–arousal cycle of each tissue (Figure 1D).

### Antioxidant Defenses

The non-enzymatic antioxidant capacity of each tissue was evaluated by the percent ABTS^+^ oxidation and uric acid concentrations. Two-fold higher ABTS^+^ oxidation was observed in the digestive gland, compared to the gill for each experimental set, while ABTS^+^ oxidation in the lung from control animals was 3-fold higher than the gill. Comparing the levels across activity, estivation and arousal groups for each tissue, the digestive gland was the only tissue that showed a significant increase in the percent ABTS^+^ oxidation during estivation and arousal, compared to control group (Ctrl = 73.6 ± 2.8; Est = 96.9 ± 5.2; and Ar = 92.2 ± 5.1%; Figure 1E). Otherwise, due to the intracellular uric acid deposits found in the digestive gland and the lung, the concentrations in these tissues were approximately 10-fold higher than those observed in the gill (Figure 1F). In the studied conditions, uric acid concentration only increased significantly in the gill of arousal group (Ctrl = 73.4 ± 7.2; Est = 93.7 ± 19.0; and Ar = 159.3 ± 26.9 mM/mg; Figure 1F).

According to our hypothesis, the response to short-term estivation and the rapid reactivation induced by early arousal induces an enzyme-mediated antioxidant response. SOD activity was always higher in the digestive gland and gill compared to the lung, but the levels went from 4-fold to 8-fold higher activity between tissues in the control active animals and the SOD activity of estivation and arousal animals, respectively. Furthermore, the digestive gland showed a significant increase in SOD activity during estivation, and a significant decrease in the arousal (Ctrl = 9.5 ± 1.9; Est = 17.5 ± 1.4; and Ar = 11.5 ± 1.8 U/mg; Figure 1G), meanwhile, in the gill and lung no significant changes were observed during the activity–estivation–arousal cycle. CAT activity was around 8-fold higher in the digestive gland than in the gill and lung. During the activity–estivation–arousal cycle, the CAT enzyme activity increased during estivation and arousal in the digestive gland (Ctrl = 78.5 ± 6.3; Est = 164.5 ± 22.8; and Ar = 133.8 ± 16.5 U/mg; Figure 1H). Also, in the gill, CAT increased its activity after estivation and decreased significantly in the arousal (Ctrl = 14.8 ± 1.8; Est = 35.9 ± 3.2; and Ar = 17.0 ± 0.9 U/mg; Figure 1H). Finally, in the lung, the animals showed a significant increase in CAT activity during estivation and arousal (Ctrl = 5.6 ± 0.7; Est = 15.1 ± 1.3; and Ar = 14.9 ± 3.1 U/mg; Figure 1H). On the other hand, GST enzyme activity in the digestive gland was 3-fold higher than in the gill and the lung. The GST activity showed a significant decrease in the digestive gland from estivation and arousal groups, compared to control (Ctrl = 573.6 ± 96.8; Est = 305.4 ± 23.6; and Ar = 250.2 ± 43.1 mU/mg; Figure 1I). Besides, GST activity showed in lung an increase during estivation and decreased significantly in the arousal (Ctrl = 83.9 ± 15.2; Est = 115.6 ± 9.2; and Ar = 80.8 ± 5.7 mU/mg; Figure 1I). In the gill no significant changes were observed.

### REDOX-Sensitive Transcription Factors (HIF1α, FOXO, and Nrf2) Expression

To evaluate changes in the expression of the REDOX-sensitive transcription factors, specific primers were designed from the genome of *P. canaliculata* (Sun et al., 2019). HIF1α and Nrf2 transcription factor sequences were identified, but in the cases of FOXO, only one sequence “FOXO-like” was found. For this reason, an *in silico* phylogenetic analysis of FOXO was previously made (methodological information is available as a Supplementary Material). The unrooted ML tree showed sequences of FoxO1-like from chordates in a basal position, locating FoxO3-like sequences of mollusks (*P. canaliculata*’s FOXO and FOXO3 from the bivalve *Sinonovacula constricta*) outside chordate FoxO3-like sequences; FoxO4-like and FoxO6 sequences were placed as derivative loci. This result showed that the FOXO locus from invertebrates is in a basal position compared to vertebrate FOXO 3, 4, and 6 domains loci (Supplementary Figure 1).

Table 1 showed an increase in FOXO expression in the studied tissues. In the gill and digestive gland from estivating snails, FOXO increased respectively its expression around 6/5-fold than control snails, and then decreased in aroused snails. In the lung of estivated snails, the increase was significantly higher than in control group (around 85 times) and remained high in aroused snails.

HIF1α and Nrf2 expression decreased significantly in the gill of *P. canaliculata* after estivation, compared to the control group. Likewise, the expression of both genes in the lung did not show significant changes during the activity–estivation–arousal cycle. Also, the expression of HIF1α did not show significant changes in the experimental groups of the digestive gland during the activity–estivation–arousal cycle, while the expression levels of Nrf2 were not detectable.

## Discussion

Distantly-related animal species have evolved adaptive strategies to tolerate environmental heat or lack of water (Storey, 2002). Estivation is a hypometabolic process that involves lowering body mass (often by dehydration), a low (or null) metabolic rate, and low oxygen availability (Navas and Carvalho, 2010; Storey and Storey, 2012). Furthermore, tissue reoxygenation during arousal from estivation induces an acute oxidative stress by ROS increase, without sufficient antioxidant defenses to neutralize them (Storey, 2002; Storey and Storey, 2010; Staples, 2016). In this scenario, some animals activate a stress-responsive physiological adaptation (the POS strategy) to cope with oxidative damage (Hermes-Lima and Storey, 1995; Giraud-Billoud et al., 2019); if this does not happen, many macromolecules can get damaged by ROS, which, in turn, can lead to cell death (Schieber and Chandel, 2014; Sies, 2015; Sies et al., 2017).

Mollusks have been proposed as model organisms to study the POS strategy after estivation; however, freshwater gastropods have not received attention comparable to terrestrial gastropods (Hermes-Lima and Storey, 1995; Hermes-Lima et al., 1998; Ramos-Vasconcelos and Hermes-Lima, 2003; Nowakowska et al., 2009; Giraud-Billoud et al., 2011, 2013; Nowakowska et al., 2011). The freshwater *P. canaliculata* is an obligate air-breather that ventilates mainly or solely with lung when dwelling in poorly oxygenated waters or burying in the mud, closing tightly its operculum at estivation (Cowie, 2002; Hayes et al., 2015). During long-term estivation (45 days), snails increase concomitantly non-enzymatic antioxidant defenses (particularly, uric acid) with TBARS levels, but without changes in the antioxidant enzymatic (CAT and SOD) defense system (Giraud-Billoud et al., 2011, 2013). A recent proteomic study focused on hypoxia tolerance showed that *P. canaliculata* is more tolerant to acute hypoxia than *Pomacea diffusa*, and it is related to a metabolic suppression and conservation of cellular fuels for extending the animal survival time under hypoxia (Mu et al., 2018). These results are consistent with the non-significant changes observed in tissue lactate concentrations of *P. canaliculata* exposed to a short-term estivation (Figure 1) and with the presence of this species in water bodies that dry seasonally or that have low oxygen concentration (Kwong et al., 2008; Hayes et al., 2015). On the other hand, a short-term estivation (7 days) induced a concomitant increase of TBARS and SOD and CAT enzyme activities (SOD only for digestive gland) without significant changes in the uric acid concentration (Figure 1).

**Figure 2:**
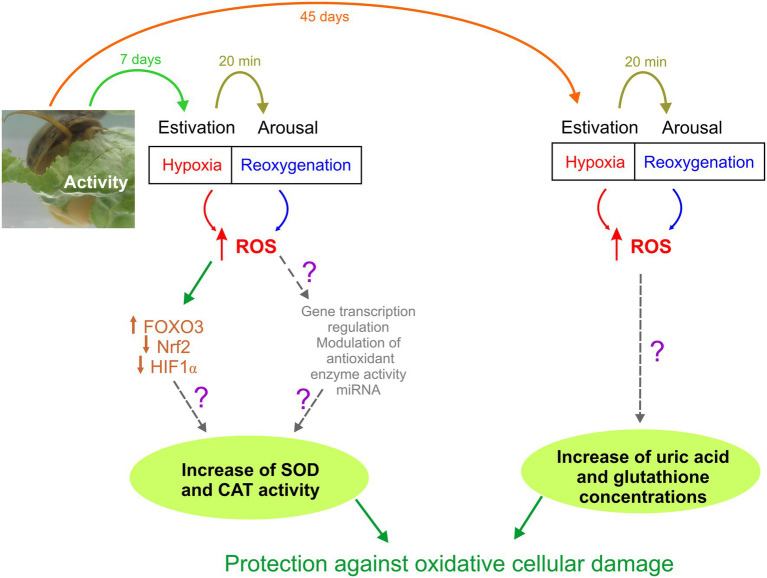
Schematic representation of *Pomacea canaliculata* responses to different periods of estivation (7 and 45 days). Unknown mechanisms have been represented with a question mark.

The gill and the lung of *P. canaliculata* are physiologically related organs that share vasculature and innervation allowing the alternation between breathing in the water and the air (Rodriguez et al., 2019, 2021). Oxidative damage of gill’s proteins and lipids by ROS (Figure 1) was evident in estivating and aroused snails, being the oxidative burst partially compensated by an increase in the CAT activity. On the other hand, there was a concomitant increase in protein damage and CAT activity in the lung of estivating and aroused animals. The greater difficulty of the gill, compared to the lung, to protect itself from oxidative burst after estivation may be related to the following facts: (a) the gill has a mitochondria rich epithelium and suffers a total collapse and dehydration during estivation (out of water), and (b) the lung may maintain some activity mobilizing variable volumes of air with each insufflation of the cavity and also has a highly-developed urate tissue (Giraud-Billoud et al., 2008). In fact, urate concentration in the lung were an order of magnitude higher than that in the gill (Figures 1, 2), suggesting that the former may access directly to this antioxidant molecule (Becker, 1993), while the latter could access only soluble uric acid from hemolymph through the slow (null) microcirculation.

The digestive gland of *P. canaliculata* is a key organ that participates in multiple and diverse physiological processes (Cowie, 2002; Hayes et al., 2015), and contains a bacterial symbiont in the digestive epithelial cells with detoxification and digestive functions (Castro-Vazquez et al., 2002; Vega et al., 2005, 2006, 2007; Godoy et al., 2013; Campoy-Diaz et al., 2018, 2020; Escobar-Correas et al., 2019). Compared to the respiratory organs, the digestive gland showed higher levels of ROS, equivalent levels of protein and lipid damage, and high SOD and CAT activities in estivating and aroused snails. Also, the digestive gland and lung showed high uric acid concentration possibly associated with the storing of urate crystalloids in the perivascular tissue (Giraud-Billoud et al., 2008). These findings indicate that the digestive gland is able to tolerate the oxidative burst induced by the activity–estivation–arousal cycle through the action of a robust defense system based on a combination of enzymatic and non-enzymatic antioxidant defenses.

Furthermore, we found a tissue and experimental condition dependent correlation between antioxidant enzymes activities and REDOX-sensitive transcription factors expression (FOXO, Nrf2, and HIF1α), in the activity–estivation–arousal cycle of *P. canaliculata*. FOXO expression and CAT activity increased in all the studied tissues of estivating snails. SOD activity changed differentially in each tissue from control and estivated snails, with an increase in SOD activity only in the digestive gland of estivated snails. Nrf2 and HIF1α expression were downregulated in the gill of estivating and aroused snails, but HIF1α showed a tendency to increase its levels during estivation in the lung and digestive gland. In this scenery, it is possible that the Nrf2 and HIFα expression in the gill is associated with their histological and functional peculiarities, i.e., the loss of gaseous exchange surface and dehydration after estivation. Future studies must clarify the inverse relationship between Nrf2 expression and antioxidant enzyme activities during the estivation of *P. canaliculata*.

In this study, we have described for the first time in a mollusk the relationship between the modification in tissue expression of transcription factors and activity of antioxidant enzymes that protect tissues exposed to a short period of hypoxia, triggered by estivation. In this sense, *P. canaliculata* is a highly resistant species to harsh environmental conditions, which makes it one of the 100 worst invasive species in the world (Lowe et al., 2000). Figure 2 shows the responses that we hypothesize for this animal model. During a short-term estivation, cells are exposed to an increase in ROS, which generates an imbalance between oxidant molecules and antioxidant defenses, potentially generating damage to macromolecules. This induces, among other potential physiological adaptive adjustments, an increase in the expression of FOXO3, which leads to an increase in the synthesis of antioxidant enzymes such as SOD and CAT that protect against oxidative stress (Figure 2, left pathway). When estivation is prolonged, as can occur in periods of drought that affect the bodies of water it inhabits, this species can also protect itself against ROS generated by non-enzymatic antioxidants such as uric acid and glutathione (Figure 2, right pathway), which allow it to extend its survival in adverse conditions, waiting the return of water.

The mechanisms that allow cells and tissues to adapt to different oxidative stress situations are a continuously growing research field. From a comparative physiology perspective, these findings may represent significant biomedical advances in the future (Hawkins and Storey, 2020). The POS strategy involves increasing antioxidant defenses before they become necessary to counteract damage from oxidative stress (Hermes-Lima et al., 1998). This anticipatory response requires finely regulated cellular and molecular mechanisms (Giraud-Billoud et al., 2019). However, there is growing evidence of interaction between different REDOX-sensitive transcription factors (Klotz et al., 2015; Klotz and Steinbrenner, 2017; Lacher et al., 2018; Gille et al., 2019), such as those studied in the experimental model proposed in this work, which could balance responses according to the cellular antioxidant demand during variable hypometabolic periods. FOXO3 appears to be the FOXO subclass present in *P. canaliculata* (Supplementary Figure 1) and their expression changes along the studied activity–estivation–arousal cycle may represent a response to enhancement of ROS during hypoxia, but also to other biological processes as the protein turnover, and cell survival and death regulation (Tzivion et al., 2011; Davy et al., 2018). Further studies could explore other associated responses of FOXO3 in *P. canaliculata* and their regulation by acetylation, ubiquitination, methylation, phosphorylation, and miRNA binding (Brown and Webb, 2018). Also, other mechanisms have been related to adjust cellular responses in animal models of estivation like epigenetic changes, such as DNA methylation that regulates the metabolism of *Apostichopus japonicus* during estivation (Zhao et al., 2015), or upregulation of miRNA like it has been described in the foot muscle of *Otala lactea* after 10-day estivation (Hoyeck et al., 2019).

The characterization of the adaptive physiological defense responses, in the face of adverse environmental conditions in animals that use the POS strategy, implies studying phenomena that are unknown until now, and therefore it is of interest in the field of comparative animal physiology.

## Data Availability Statement

The raw data supporting the conclusions of this article will be made available by the authors, without undue reservation.

## Ethics Statement

Procedures for snail culture, sacrifice, and tissue sampling were approved by the Institutional Committee for the Care and Use of Laboratory Animals (CICUAL, Facultad de Ciencias Médicas, Universidad Nacional de Cuyo), Approval Protocol No. 55/2015.

## Author Contributions

All authors listed have made a substantial, direct, and intellectual contribution to the work, and approved it for publication.

## Funding

This work was supported by grants from Ministerio de Ciencia, Tecnología e Innovación, Agencia Nacional de Promoción de la Investigación, el Desarrollo Tecnológico y la Innovación, Argentina (PICT-2018-03966-BID) and Secretaría de Investigación, Internacionales y Posgrado, Universidad Nacional de Cuyo, Argentina (Proyecto Tipo I, 06/J511).

## Conflict of Interest

The authors declare that the research was conducted in the absence of any commercial or financial relationships that could be construed as a potential conflict of interest.

## Publisher’s Note

All claims expressed in this article are solely those of the authors and do not necessarily represent those of their affiliated organizations, or those of the publisher, the editors and the reviewers. Any product that may be evaluated in this article, or claim that may be made by its manufacturer, is not guaranteed or endorsed by the publisher.
