# Supplementary material for: Antioxidant Responses Induced by Short-Term Activity–Estivation–Arousal Cycle in Pomacea canaliculata
Source: Front Physiol. 2022 Feb 2;13:805168. doi: 10.3389/fphys.2022.805168 (PMC8847974; doi:10.3389/fphys.2022.805168)
Supplement: Supplementary file 1 [file Data_Sheet_1.docx]

Supplementary Material

**Supplementary Figure 1:**

**
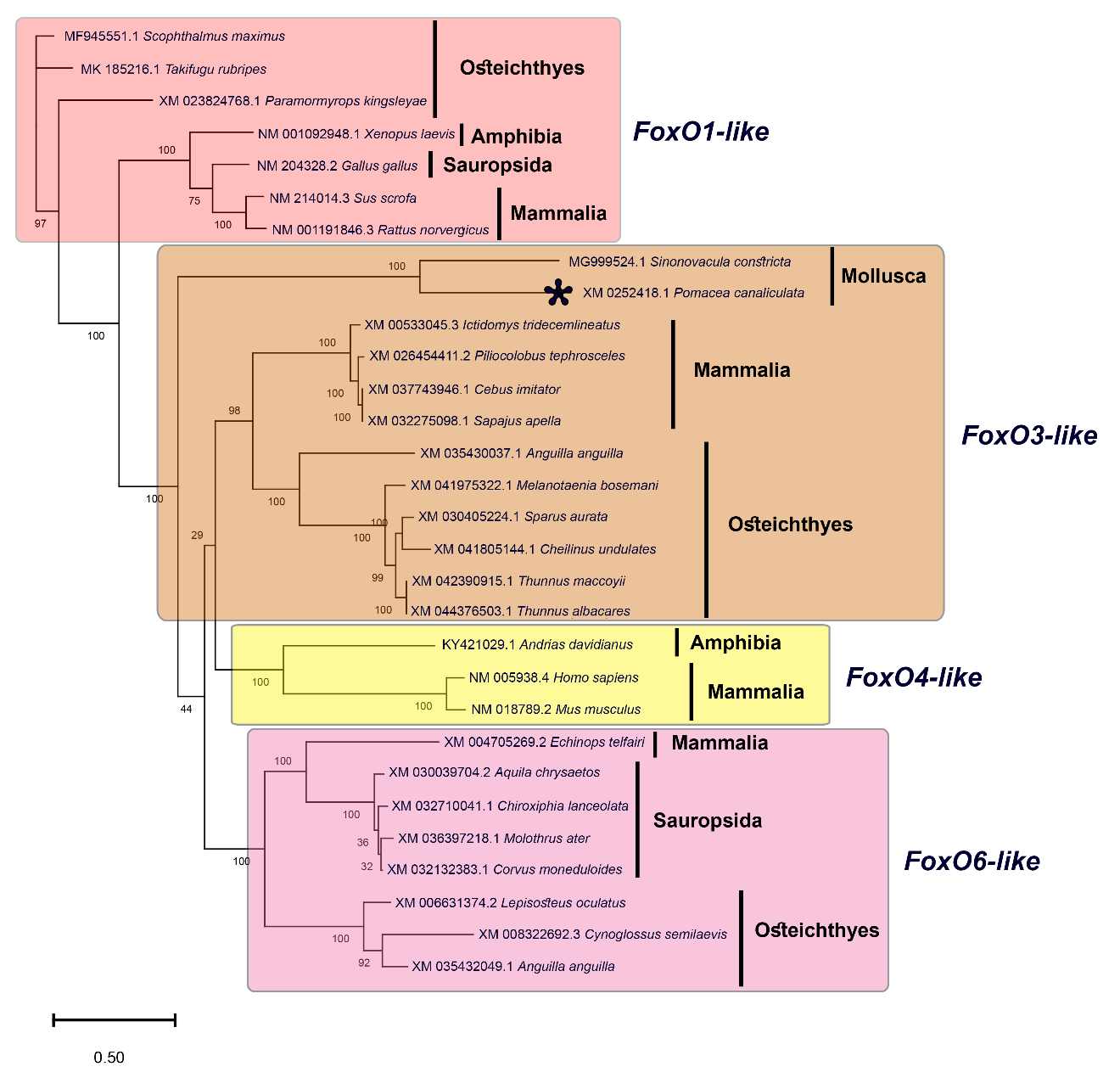
**

**Supplementary Figure 1:** Maximum Likelihood phylogenetic tree showing the evolutionary history of 30 FoxO-like (1,3,4 and 6) proteins and associated taxa. Monophyletic taxa are highlighted to the right of the topology tree. The *P.canaliculata*’s FOXO sequence is labeled with an asterisk. The bootstrap values are showed as percentage in each node. The bar represents genetic distance.

***In silico* FOXO analysis**

**a) Amplified region**

Primers (forward 5’-3’ GCTGGCTGGAAGAACTCCAT and reverse 5’-3’ ATTGATGACCCACCAGGAGC) were designed for amplifying a region of 102 bp for *Pomacea canaliculata* forkhead box protein O-like (LOC112565983; accession number XM_025241893, about 9300 bp; https://www.ncbi.nlm.nih.gov/nuccore/XM_025241893):

5’-GCTGGCTGGAAGAACTCCATCCGGCACAACCTTTCCCTTCACAGTCGCTTTATGCGAATACAAAATGAGGGAACTGGAAAGAGCTCCTGGTGGGTCATCAAT-‘3

The size of the amplicon was corroborated by standard PCR in agarose gel (1.5%), using as template cDNA from tissues of raised Lab-*P. canaliculata*. The 100 bp amplicon of FOXO is highlighted with a red rectangle:


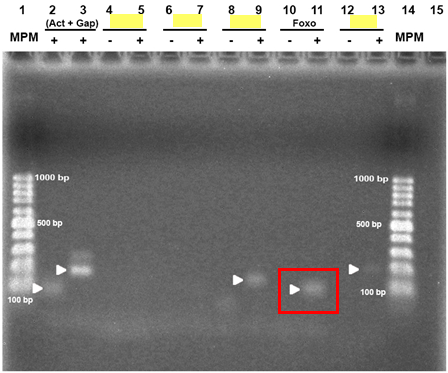


**b) BLAST analysis**

A broad, unrestricted search with BLASTn algorithm was done (<https://blast.ncbi.nlm.nih.gov/Blast.cgi?PROGRAM=blastn&BLAST_PROGRAMS=megaBlast&PAGE_TYPE=BlastSearch&SHOW_DEFAULTS=on&LINK_LOC=blasthome>) Below it showed the main hits:

*Sinonovacula constricta* (mollusk: bivalves) FOXO 3: 90.1% identity; 100% cover; **E value** 9e-29

Chordata (fishes-mammals) FOXO 3: 88.1-87.13% identity, 99% cover; **E value** 5e-26- 2e-24

Chordata (fishes) FOXO 1. 89%; 91 cover, **E value** 2e-24- 2e-23

Chordata (mammals) FOXO 3 86.14% identity, 99% cover, **E value** 2e-23.

**c) Phylogenetic analysis**

For elucidating the evolutionary history, a Maximum Likelihood tree (Supplementary Figure 1) was constructed using selected homologous sequences with an appropriate cover and similarity, which encompassed sequences of FOXO1, 3, 4, and 6 (see accession number and species in the tip of each tree branches). Initial alignments, trimming and edition was done with ClustalOmega ([Sievers et al., 2011](#_ENREF_5)) and MEGA11 ([Tamura et al., 2021](#_ENREF_6)). The final alignment (30 nucleotide sequences, 4063-963 sites input) was done with MAFFT v7.490, E-INS-I algorithm) ([Katoh and Standley, 2013](#_ENREF_3);[Katoh et al., 2017](#_ENREF_2)). The final phylogenetic inference was done with PhyML ([Guindon et al., 2010](#_ENREF_1)) (Maximum Likelihood method); the model of substitution that best fit with our set of data (HKY85) was obtained with Smart Model Selection ([Lefort et al., 2017](#_ENREF_4)) from PhyML package. The tree with the highest log-likelihood value (-63506.85484) was chosen. A bootstrap analysis with 100 repetitions was done.

**Supplementary Table 1:** RT-PCR specific primers and characteristics of amplicons. FOXO (forkhead box protein O-like), HIF1α (hypoxia-inducible factor 1-alpha), Nrf2 (nuclear factor erythroid 2).

| **Gene** | **N° GenBank**  **access** | **Sense (**5´🡪 3´) | **Antisense (**5´🡪 3´) | **Amplicon** | |
| --- | --- | --- | --- | --- | --- |
|  |  |  |  | **Size (bp)** | **Tm (°C)** |
| FOXO | XM_025241893.1 | GCTGGCTGGAAGAACTCCAT | ATTGATGACCCACCAGGAGC | 102 | 82 |
| HIF1α | XM_025236476.1 | TGACAGACAAAGCCAGGTTGA | ATTGCTTGCTGACCCCGTAA | 84 | 80 |
| Nrf2 | XM_025226179.1 | GAACGAGAACGGGAGCTTGA | ATGGCACCCATTCACCTGTT | 145 | 81.5 |
| Beta-Actin | KM504520.1 | TCACCATTGGCAACGAGCGAT | TCTCGTGAATACCAGCCGACT | 87 | 83.5 |

**Supplementary Table 2:** ANOVA table showing F and P values of each compound comparison during the activity-estivation-arousal cycle and between tissues (ANOVA, Newman-Keuls´s test).

|  | F (Dfn, Dfd)= | P= |
| --- | --- | --- |
| Reactive oxygen species (ROS)  -Cycle comparison-Digestive gland | F (2, 8) = 5,910 | P=0,0265 |
| -Cycle comparison-Gill | F (2, 9) = 15,81 | P=0,0011 |
| -Cycle comparison-Lung | F (2, 6) = 1,720 | P=0,2568 |
| -Tissue comparison activity group | F (2, 7) = 728,8 | P<0,0001 |
| -Tissue comparison estivation group | F (2, 8) = 57,70 | P<0,0001 |
| -Tissue comparison arousal group | F (2, 8) = 71,78 | P<0,0001 |
| Protein oxidative damage (carbonyl groups, CG)  -Cycle comparison-Digestive gland | F (2, 9) = 7,823 | P=0,0107 |
| -Cycle comparison-Gill | F (2, 9) = 21,86 | P=0,0004 |
| -Cycle comparison-Lung | F (2, 9) = 40,12 | P<0,0001 |
| -Tissue comparison activity group | F (2, 9) = 51,28 | P<0,0001 |
| -Tissue comparison estivation group | F (2, 9) = 18,45 | P=0,0007 |
| -Tissue comparison arousal group | F (2, 9) = 13,33 | P=0,0020 |
| Thiobarbituric acid reactive substances (TBARS)  -Cycle comparison-Digestive gland | F (2, 12) = 9,914 | P=0,0029 |
| -Cycle comparison-Gill | F (2, 10) = 6,677 | P=0,0144 |
| -Cycle comparison-Lung | F (2, 12) = 1,231 | P=0,3263 |
| -Tissue comparison activity group | F (2, 11) = 29,77 | P<0,0001 |
| -Tissue comparison estivation group | F (2, 12) = 34,11 | P<0,0001 |
| -Tissue comparison arousal group | F (2, 11) = 39,23 | P<0,0001 |
| Lactate concentration  -Cycle comparison-Digestive gland | F (2, 8) = 1,988 | P=0,1991 |
| -Cycle comparison-Gill | F (2, 9) = 0,8735 | P=0,4501 |
| -Cycle comparison-Lung | F (2, 9) = 3,380 | P=0,0804 |
| -Tissue comparison activity group | F (2, 6) = 5,446 | P=0,0448 |
| -Tissue comparison estivation group | F (2, 11) = 11,61 | P=0,0019 |
| -Tissue comparison arousal group | F (2, 9) = 5,564 | P=0,0267 |
| Percent of ABTS^+^ oxidation  -Cycle comparison-Digestive gland | F (2, 11) = 6,323 | P=0,0149 |
| -Cycle comparison-Gill | F (2, 11) = 0,3613 | P=0,7048 |
| -Cycle comparison-Lung | F (2, 12) = 3,608 | P=0,0593 |
| -Tissue comparison activity group | F (2, 11) = 128,4 | P<0,0001 |
| -Tissue comparison estivation group | F (2, 11) = 44,25 | P<0,0001 |
| -Tissue comparison arousal group | F (2, 12) = 45,51 | P<0,0001 |
| Uric acid concentration  -Cycle comparison-Digestive gland | F (2, 12) = 1,404 | P=0,2832 |
| -Cycle comparison-Gill | F (2, 11) = 5,502 | P=0,0221 |
| -Cycle comparison-Lung | F (2, 10) = 0,9534 | P=0,4178 |
| -Tissue comparison activity group | F (2, 10) = 13,00 | P=0,0017 |
| -Tissue comparison estivation group | F (2, 11) = 12,83 | P=0,0013 |
| -Tissue comparison arousal group | F (2, 12) = 21,61 | P=0,0001 |
| SOD activity  -Cycle comparison-Digestive gland | F (2, 10) = 5,231 | P=0,0279 |
| -Cycle comparison-Gill | F (2, 10) = 2,832 | P=0,1060 |
| -Cycle comparison-Lung | F (2, 11) = 1,370 | P=0,2942 |
| -Tissue comparison activity group | F (2, 10) = 9,349 | P=0,0051 |
| -Tissue comparison estivation group | F (2, 9) = 45,50 | P<0,0001 |
| -Tissue comparison arousal group | F (2, 12) = 25,80 | P<0,0001 |
| CAT activity  -Cycle comparison-Digestive gland | F (2, 12) = 6,868 | P=0,0103 |
| -Cycle comparison-Gill | F (2, 11) = 30,49 | P<0,0001 |
| -Cycle comparison-Lung | F (2, 12) = 7,442 | P=0,0079 |
| -Tissue comparison activity group | F (2, 12) = 107,5 | P<0,0001 |
| -Tissue comparison estivation group | F (2, 11) = 33,50 | P<0,0001 |
| -Tissue comparison arousal group | F (2, 12) = 49,23 | P<0,0001 |
| GST activity  -Cycle comparison-Digestive gland | F (2, 8) = 9,257 | P=0,0083 |
| -Cycle comparison-Gill | F (2, 10) = 0,5251 | P=0,6070 |
| -Cycle comparison-Lung | F (2, 9) = 4,499 | P=0,0442 |
| -Tissue comparison activity group | F (2, 7) = 22,68 | P=0,0009 |
| -Tissue comparison estivation group | F (2, 10) = 14,57 | P=0,0011 |
| -Tissue comparison arousal group | F (2, 10) = 8,626 | P=0,0067 |

**References**

Guindon, S., Dufayard, J.-F., Lefort, V., Anisimova, M., Hordijk, W., and Gascuel, O. (2010). New algorithms and methods to estimate maximum-likelihood phylogenies: assessing the performance of PhyML 3.0. *Systematic Biology* 59**,** 307-321.

Katoh, K., Rozewicki, J., and Yamada, K. (2017). MAFFT online service: multiple sequence alignment, interactive sequence choice and visualization. *Briefings in Bioinformatics* 20**,** 1160-1166.

Katoh, K., and Standley, D. (2013). MAFFT multiple sequence alignment software version 7: improvements in performance and usability. *Molecular Biology and Evolution* 30**,** 772-780.

Lefort, V., Longueville, J.-E., and Gascuel, O. (2017). SMS: Smart model selection in PhyML. *Molecular Biology and Evolution* 34**,** 2422-2424.

Sievers, F., Wilm, A., Dineen, D., Gibson, T., Karplus, K., Li, W., Lopez, R., Mcwilliam, H., Remmert, M., and Söding, J. (2011). Fast, scalable generation of high-quality protein multiple sequence alignments using Clustal Omega. *Molecular Systems Biology* 7**,** 539.

Tamura, K., Stecher, G., and Kumar, S. (2021). MEGA11: Molecular Evolutionary Genetics Analysis Version 11. *Molecular Biology and Evolution* 38**,** 3022-3027.
